# Design and Experiment of an Unoccupied Control System for a Tracked Grain Vehicle

**DOI:** 10.3390/s24092715

**Published:** 2024-04-24

**Authors:** Jiahui Pan, Lizhang Xu, En Lu, Buwang Dai, Tiaotiao Chen, Weiming Sun, Zhihong Cui, Jinpeng Hu

**Affiliations:** Agricultural Engineering School, Jiangsu University, Zhenjiang 212013, China; 212116050@stmail.ujs.edu.cn (J.P.); jsluen@ujs.edu.cn (E.L.); 2222116061@stmail.ujs.edu.cn (B.D.); 2111916011@stmail.ujs.edu.cn (T.C.); 2112316034@stmail.ujs.edu.cn (W.S.); 2212216077@stmail.ujs.edu.cn (Z.C.); hujinpeng@stmail.ujs.edu.cn (J.H.)

**Keywords:** tracked grain vehicle, automatic navigation, pure pursuit algorithms, automatic grain unloading, grain tank recognition

## Abstract

In order to enhance crop harvesting efficiency, an automatic-driving tracked grain vehicle system was designed. Based on the harvester chassis, we designed the mechanical structure of a tracked grain vehicle with a loading capacity of 4.5 m^3^ and a grain unloading hydraulic system. Using the BODAS hydraulic controller, we implemented the design of an electronic control system that combines the manual and automatic operation of the chassis walking mechanism and grain unloading mechanism. We utilized a hybrid A* algorithm to plan the traveling path of the tracked grain vehicle, and the path-tracking controller of the tracked grain vehicle was designed by combining fuzzy control and pure pursuit algorithms. Leveraging binocular vision technology and semantic segmentation technology, we designed an automatic grain unloading control system with functions of grain tank recognition and grain unloading regulation control. Finally, we conducted experiments on automatic grain unloading control and automatic navigation control in the field. The results showed that both the precision of the path-tracking control and the automatic unloading system meet the requirements for practical unoccupied operations of the tracked grain vehicle.

## 1. Introduction

As the final stage of grain production, the harvest operation requires swift and efficient collection to capitalize on favorable agricultural opportunities. However, during the harvest process, the combine harvester’s grain tank has limited capacity, necessitating drives to the edge of the field to unload grain when it becomes full. This unloading procedure consumes a significant amount of non-harvesting time for the combine harvester, thereby greatly affecting crop harvesting efficiency [1,2]. Taking the WORLD Company’s combine harvester (with a grain tank volume of 1.6 m^3^) for rice harvesting as an example, assuming that the operating efficiency is 0.53 Hm^2^/h, with 10 h of harvesting per day, a yield of 40 kg/ Hm^2^, and a bulk density of 600 kg/m^3^, each time when the grain tank is full, it is necessary to unload the grain 50 times per day. With each unloading event taking approximately 3 min, the combine harvester spends at least 2.5 h a day on unloading. Therefore, implementing a cooperative unloading method between grain transport vehicles and combine harvesters can effectively reduce the non-harvesting time spent by the combine harvester, which is vital for improving the efficiency of crop harvesting [3].

Existing grain transport vehicles are mainly wheeled vehicles or wheeled tractors that haul grain containers [4], but most of the rice harvesting operations are predominantly carried out in soft and muddy fields, where wheeled vehicles are prone to skidding and sinking, which renders normal cooperative operations impossible. On the contrary, a tracked chassis possesses advantages such as low ground pressure and excellent pass ability, so the tracked grain vehicle is better suited for transportation tasks in small and medium-sized wet and muddy rice fields. Based on the exclusive version of the combine harvester chassis of the WORLD Company, we designed an unoccupied autonomous tracked grain transport vehicle to transfer the grain from the combine harvester to the transportation vehicle on the agricultural road through unloading co-operation and final transport to the grain depot, thereby enhancing the efficiency of rice harvesting operations.

At present, the research on the automatic control of autonomous tracked agricultural machinery mainly focuses on tractors, seeders, rice harvesters, and other agricultural machines [5,6,7], such as the use of throttle control mechanisms, electro-hydraulic walking systems, and navigation tracking controllers to achieve the automatic driving of tractors [8,9]; GPS navigation systems, speed sensors, automatic speed control mechanisms, and industrial computers to achieve the automatic driving operation of combine harvesters [10,11]; and the use of GPS speed measurement, ground wheel encoders, closed-loop stepping motors, and double-guide sliding deflection devices to achieve the operation control system of seeders [12,13,14,15], but there is still no research reports on tracked grain vehicles in this area. Existing grain tank recognition technologies mainly include edge extraction technology based on monocular cameras, which determine the filling status of the grain tank by assessing the contact between the grain’s envelope and the tank’s edges [16]. Additionally, there are 3D point cloud techniques based on the fusion of multiple cameras, which establish a 3D model to obtain the complete loading state by filling the point cloud [17]. The existing research primarily focuses on identifying the loading status of the grain tank, with few research achievements utilizing visual recognition results to guide automatic control for unloading processes.

In response to the mentioned challenges, based on the exclusive version of the combine harvester chassis of the WORLD Company, a tracked grain vehicle with a loading capacity of 4.5 m^3^ was designed, an electronic control system that combines manual and automatic operation for the chassis’ walking and unloading mechanisms was constructed based on the BODAS hydraulic controller, an unoccupied driving control system for the tracked grain vehicle was designed combining the hybrid A* algorithm and the fuzzy adaptive pure pursuit control method, and the grain tank recognition and automatic unloading control system were designed using binocular vision semantic segmentation technology. To ensure accuracy and reliability, extensive field experiments were conducted to validate the unoccupied driving and automatic unloading systems of the tracked grain vehicle.

## 2. Materials and Methods

### 2.1. Design of the Control System for the Tracked Grain Vehicle

**Figure 1:**
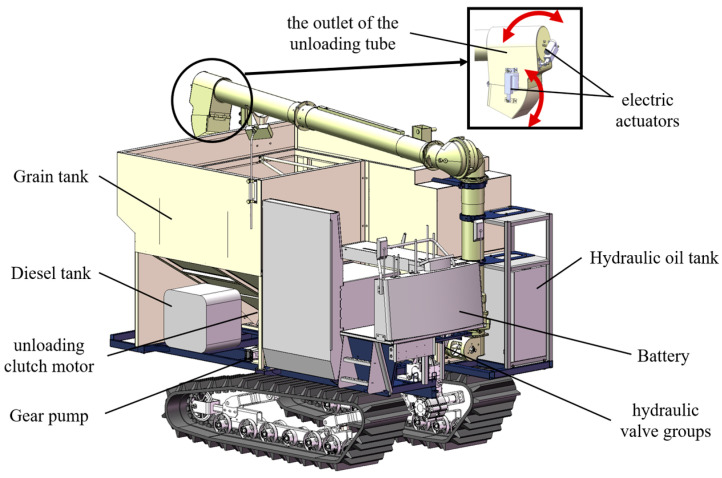
Design diagram of the tracked grain vehicle.

Based on the exclusive version of the combine harvester chassis of the WORLD Company, an unoccupied tracked grain vehicle with a 4.5 m^3^ loading capacity was specifically designed in Figure 1. To maximize space utilization, the grain tank featured a V-shaped design with a wider upper section and a narrower lower section. This design facilitated the installation of hydraulic components such as the battery, diesel tanks, unloading clutch motor, hydraulic valve groups, hydraulic filters, gear pumps, and other hydraulic parts while optimizing the grain tank’s volume. The size of the grain tank determined the design of an elongated and thickened horizontal grain-stirring auger, which was regulated by a hydraulic motor to adjust the stirring speed of the auger. Precise control over the unloading location was achieved through the use of two electric actuators governing the outlet of the unloading tube rotation with two degrees of freedom.

#### 2.1.1. Design of the Electrical Control System

**Figure 2:**
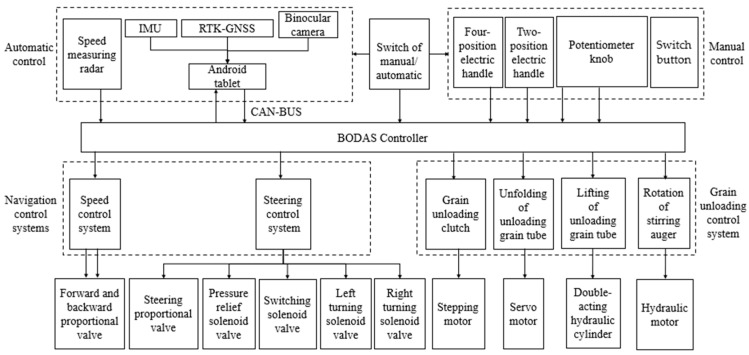
Overall design schematic of the control system for the tracked grain vehicle.

The electrical control system for the unoccupied tracker grain vehicle was developed using an electric control lever (analog voltage operating lever), a three-position and four-way proportional valve group, and a BODAS controller (illustrated in Figure 2), in which the bottom controller (Rexroth BODAS controller RC28-14, Bosch Rexroth, Shanghai, China) received the electrical signals of the electric control lever, the switch button, and knob or the CAN signal from the Android tablet to regulate the movement and unloading systems of the tracked grain vehicle. By adjusting the opening and closing of the hydraulic valve groups in the chassis, the vehicle achieved straight-line variable speed and steering mode switching by adjusting the opening of the unloading hydraulic valve group. The elevation control of the unloading tube and the rotation control of the grain unloading stirring auger were accomplished by adjusting the aperture of the hydraulic valve assembly. Moreover, the unfolding of the unloading tube was achieved through simultaneous control of the stepping motor and the servo motor.

#### 2.1.2. Electrical Control Design of the Chassis Walking Mechanism

**Figure 3:**
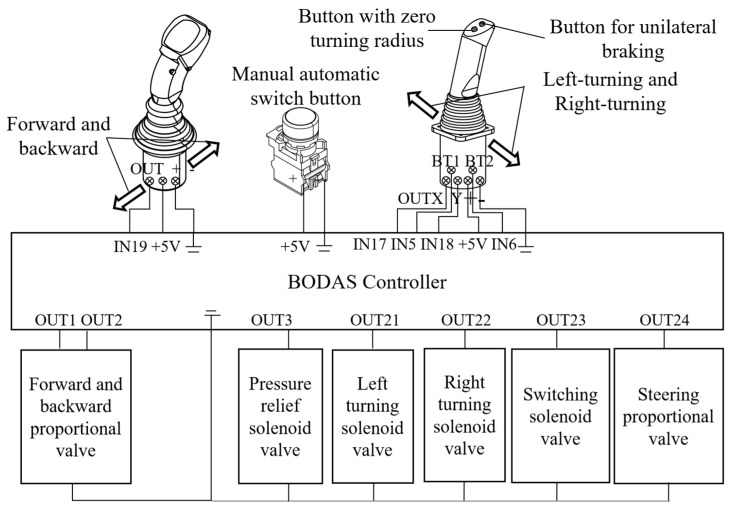
Electrical control structure of the grain vehicle’s walking mechanism.

The chassis walking mechanism, based on the harvester chassis, incorporated a straight-line speed control mechanism and a left–right steering mechanism. The electrical control structure for this mechanism is illustrated in Figure 3. The two-position electric handle outputted a 0–5 V analog voltage signal to the BODAS controller, which regulated the opening degree of the proportional valve for forward and backward control and controlled the rotating shaft of the HST variable displacement hydraulic pump to achieve speed adjustment. Additionally, the four-position electric handle delivered a 0–5 V analog voltage signal to the BODAS controller, which emitted an analog current to control the opening degree of the steering proportional valve and at the same time outputted four switching quantities that governed four solenoid valves responsible for left turns, right turns, pressure relief, and switching, of which the left- and right-turning solenoid valves controlled the left and right steering clutch. The pressure relief valve opened during steering and closed during straight-line travel, and the switching solenoid valves, in combination with the steering proportional valve, enabled three steering modes: situ steering, differential steering, and one-sided braking steering. In differential steering mode, the current value of the steering proportional valve varied inversely with the turning radius. Apart from manual control facilitated by the aforementioned lever handles, automatic control was attainable by pressing the manual/automatic switch. In automatic control mode, the BODAS controller received control commands from the Android tablet via the CAN bus and controlled the opening of the proportional valve and the on/off state of the solenoid valve for the tracked grain vehicle’s forward and backward motion and steering.

#### 2.1.3. Electro-Hydraulic Design of the Grain Unloading Mechanism

**Figure 4:**
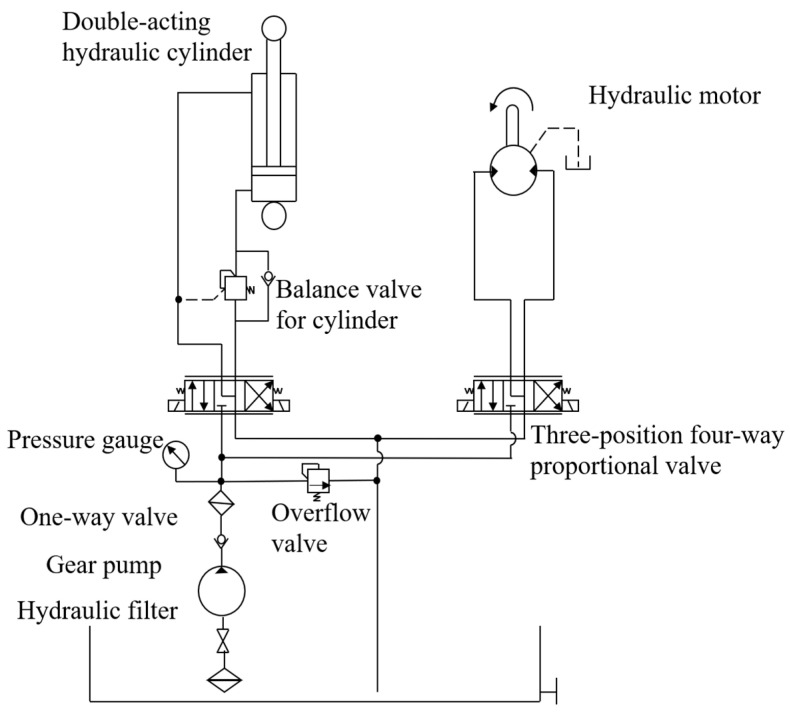
Grain unloading hydraulic system diagram of the grain vehicle.

The engine speed was transferred to the gear pump via a belt during the grain unloading process; the hydraulic system is illustrated in Figure 4. It comprised a pulley for the gear pump with a diameter of 170 mm and a pulley for the output shaft of the engine with a diameter of 210 mm; the transmission ratio between these pulleys was 17:21. Consequently, when the engine reached its maximum speed of 2500 rpm, the gear pump rotated at 3088 rpm. To fulfill the requirements, the Rexroth AZPG-22-40OLDC gear pump (Bosch Rexroth, Shanghai, China) was selected, featuring a displacement of 40 mL/r and a maximum flow rate of 123 L/min. Two sets of proportional valves received an analog current (4–20 mA) to regulate the double-acting hydraulic cylinder and hydraulic motors, respectively, and the maximum flow rates of the valves were 10 L/min and 65 L/min. Meanwhile, the double-acting hydraulic cylinder, responsible for controlling the lifting and lowering of the grain unloading tube, managed a maximum flow rate of 10 L/min and had a 200 mm stroke. In order to ensure that the rotational speed of 800 rpm for the grain unloading stirring auger, the hydraulic motor necessitated a minimum flow rate of 65 L/min, so the chosen hydraulic motor, belonging to the BMSY series from Zhenjiang Dali Hydraulic Motor Company, had a displacement of 80.6 mL/r.

**Figure 5:**
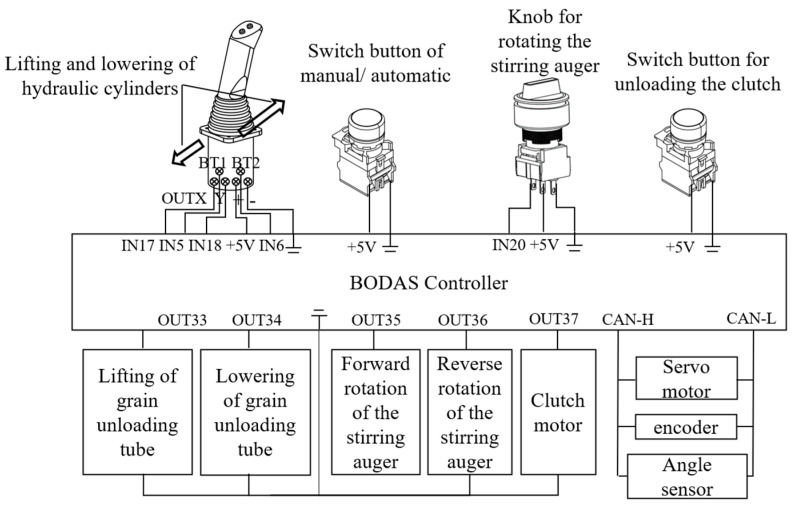
Electrical control structure of the grain vehicle’s unloading mechanism.

The integrated device that combined manual and automatic options for the grain unloading system encompassed the grain unloading clutch button, grain unloading clutch motor, electric control lever, potentiometer knob, double-acting hydraulic cylinder, two sets of three-position four-way proportional valves, hydraulic motor, and grain unloading motor; the electronic control structure is displayed in Figure 5. The grain unloading function included unfolding and lifting the grain unloading tube, rotating the grain unloading stirring auger, and engaging the grain unloading clutch. The unfolding of the grain unloading tube was accomplished by controlling a servo motor (Keiou Motor 90WSCB-001, Shandong Kaiou Motor Tech Co., Zibo, China), the lifting of the grain unloading tube and the rotation of the grain unloading stirring auger were driven by adjusting the opening of the two sets of three-position four-way proportional valves to extend or retract the hydraulic cylinder and rotate the hydraulic motor, respectively, and the grain unloading clutch function was executed by a stepper motor that tightened or loosened the transmission belt, thereby activating the articulated tensioning wheel to engage or disengage the clutch. The servo motor’s encoder provided feedback on the motor’s rotation angle, while an angle sensor installed on the grain unloading tube offered feedback on the extension and retraction of the double-acting hydraulic cylinder, enabling closed-loop control.

#### 2.1.4. Design of the Automatic Operation Control System

**Figure 6:**
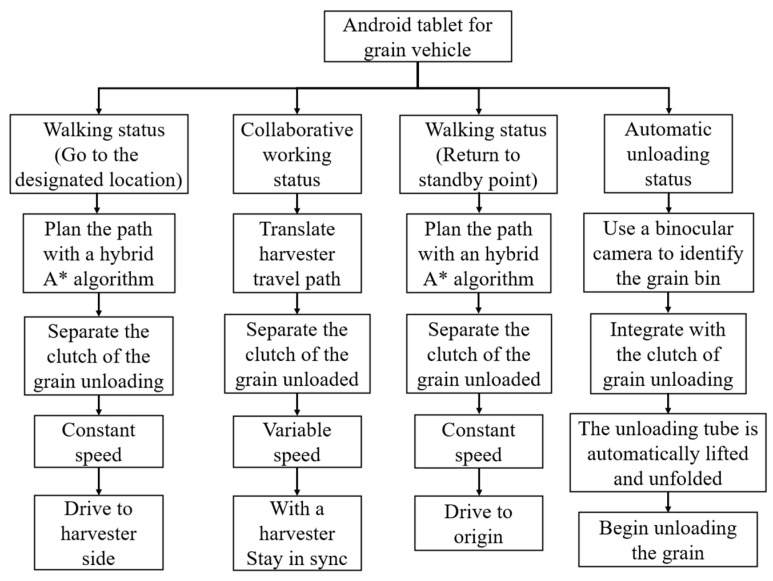
Control logic of grain vehicle.

According to the operational requirements of the collaborative operation between the tracked grain vehicle and the combine harvester, the control logic of the grain vehicle is shown in Figure 6. When the loading rate of the combine harvester grain bin reached 90%, it transmitted a grain unloading co-operative signal and location information to the Android tablet of the grain vehicle via a 4G transmitting module. Upon receiving the signal, the grain vehicle began moving and sent the positioning signal to the combine harvester when it reached the designated location. Subsequently, the combine harvester expanded the grain unloading tube and initiated unloading while the grain vehicle maintained a consistent speed, traveling alongside. When the combine harvester’s grain tank was emptied, it sent a signal indicating the completion of unloading to the grain vehicle, and the vehicle started to return to the standby point. When the vehicle arrived at the standby point, it expanded the grain unloading tube, looked for the unloading point through the binocular camera, and then the unloading stirring auger rotated to initiate the unloading process.

### 2.2. Autonomous Navigation and Grain Unloading Control System Design

#### 2.2.1. Navigation Control System

**Figure 7:**
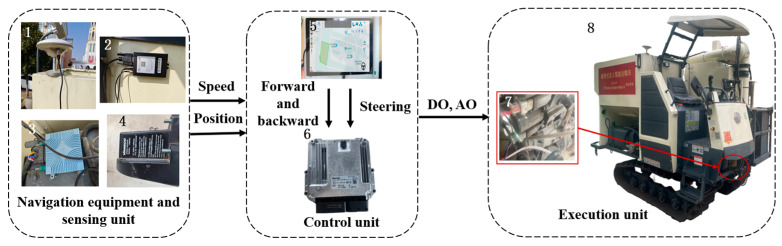
Automatic control system of the tracked grain vehicle. 1. GNSS antenna; 2. DTU; 3. BeiDou combined navigation module; 4. Speed measuring radar; 5. Android tablet; 6. Rexroth hydraulic controller; 7. Chassis travelling hydraulic valve group; 8. Tracked grain vehicle.

The navigation control system of the tracked grain vehicle was primarily composed of navigation devices, sensor units, a control unit, and an execution unit, as depicted in Figure 7. The sensor units comprised a GNSS antenna, a BeiDou combined navigation module (MS6111), DTU (MD649), and speed measuring radar (MICRO-TRAK) (Beijing BDStar Navigation Co. Ltd. Beijing, China). The control unit employed the signal input pins of the Android tablet and the Bosch Rexroth BODAS hydraulic controller (Bosch Rexroth, Shanghai, China) to gather information from the sensing devices and controlled the walking module and automatic grain unloading module through the signal output pin. The executive unit consisted of the straight-line travelling system and steering system, which was composed of the chassis walking hydraulic valve group.

#### 2.2.2. Path Planning of the Grain Vehicle

**Figure 8:**
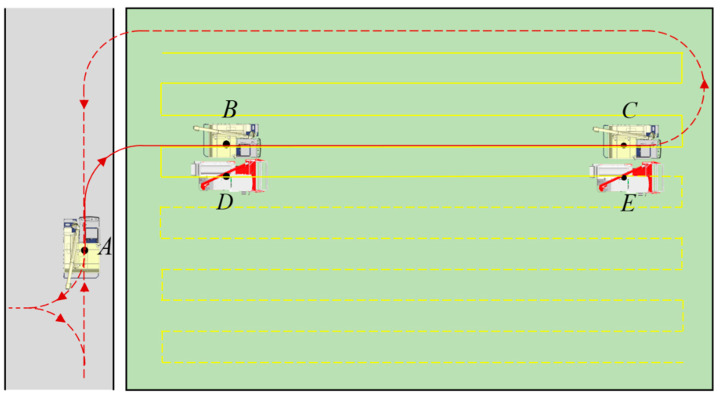
Path planning of the tracked grain vehicle.

As illustrated in Figure 8, the tracked combined harvester adopted a zigzag operation path. At point D, it was anticipated that the grain tank would be filled (>90%). Point B, defined as the unloading point, was obtained by moving the D-point vertically towards the already harvested area by the operating width of the combine harvester. The pose information of unloading point B was transmitted to the grain vehicle through the 4G transmitting module. The unharvested field was used as an obstacle, and the field environment model was combined with the hybrid A* algorithm to plan a path for the grain vehicle from point A to point B, which represented the route from the field edge to the starting position of coordinated grain unloading. After the grain vehicle reached point B according to the generated path, the two vehicles initiated coordinated grain unloading. The grain vehicle moved in sync with the combine harvester along the operating path that the harvester had already travelled until point C, where the coordinated unloading was completed. Subsequently, the grain vehicle employed a hybrid A* algorithm to plan the return path and returned to point A.

#### 2.2.3. Path-Tracking Controller

**Figure 9:**
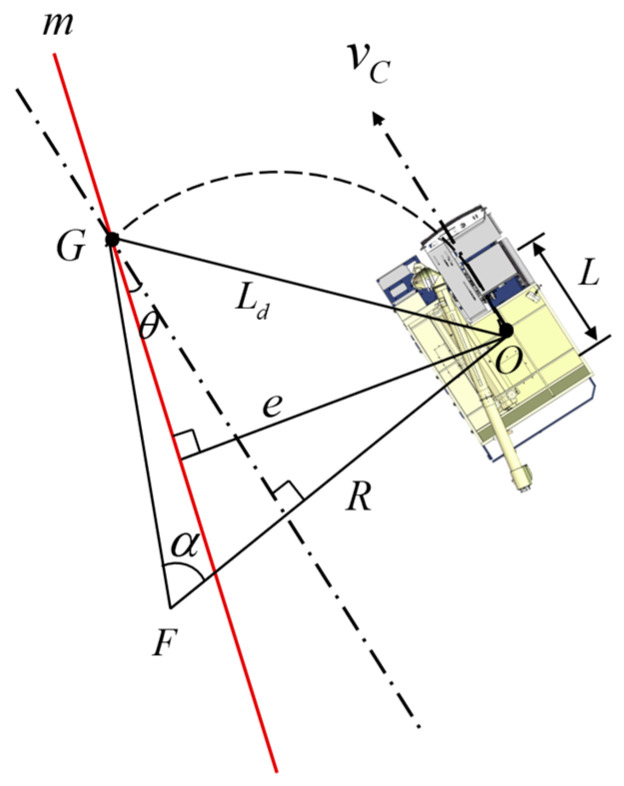
Pure pursuit model for the tracked grain vehicle.

The pure pursuit model is a geometric tracking model that plans the arc path from a vehicle’s current position to the target position based on the relationship between the cornering angle and the forward-looking distance to obtain the steering radius with the determination of the appropriate forward-looking t distance and uses the steering radius and the differential steering mode to derive the instantaneous steering angle. As shown in Figure 9, $G\left(x_{g},y_{g}\right)$ indicates the coordinates of the forward-looking point, the forward-looking distance denoted as $L_{d}$, the instantaneous steering radius represented by R, the length of the track in contact with the ground denoted as L, the target path represented by m, the lateral deviation of the vehicle from the target path at the current moment denoted as e, and θ, the angle between the vehicle’s central axis and the target path at the current moment, referred to as the heading deviation [18,19].

According to the relationship of each parameter, the relationship for the instantaneous steering radius R, lateral deviation e, heading deviation θ, and forward-looking distance $L_{d}$ is obtained as follows [20].

(1) $$R=L_{d}^{2}/(2(e\cos\theta +\sqrt{L_{d}^{2}-e^{2}}\sin\theta ))$$

By conducting a kinematic analysis of the tracked grain vehicle, we established the relationship between the instantaneous steering angle δ and steering radius R, as well as the track length in contact with the ground L.

(2) $$\delta =\tan^{-1}\left(L/R\right)$$

By combining Equations (1) and (2), we derived the relationship for the instantaneous steering angle δ, lateral deviation e, heading deviation θ, forward-looking distance $L_{d}$, and track length in contact with the ground L.

(3) $$\delta =\tan^{-1}(2L(e\cos\theta -\sqrt{L_{d}^{2}-e^{2}}\sin\theta )/L_{d}^{2})$$

**Figure 10:**
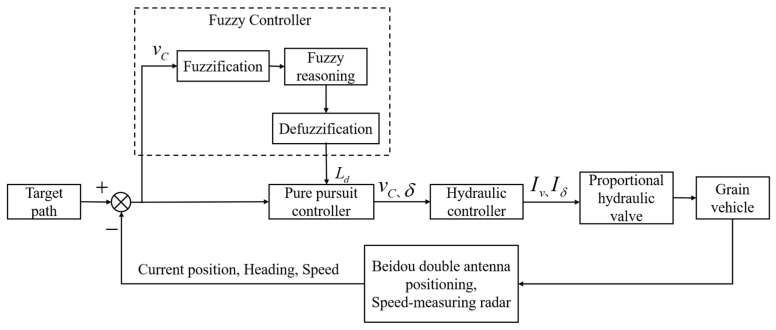
Schematic diagram of the path-tracking control system.

The pure pursuit algorithms emulate the driving habits of a human driver and obtain the target point for tracking motion based on the forward-looking distance, so the accuracy of the control depends on the value of the forward-looking distance [21]. It was found through experiments that the relationship between the forward-looking distance, vehicle speed, and the effectiveness of path-tracking control is not linear. Therefore, this study introduced fuzzy control to dynamically adjust the forward-looking distance at different vehicle speeds, aiming to enhance the stability of the path-tracking control system. Figure 10 presents the control system schematic. The forward speed $v_{C}$ and instantaneous steering angle δ output from the pure pursuit controller were linearly related to the forward proportional valve current $I_{v}$ and the steering proportional valve current $I_{\delta }$ output from the hydraulic controller, respectively.

Based on the aforementioned analysis, the vehicle speed $v_{c}$ serves as the input for the fuzzy adaptive pure pursuit controller, whereas the forward-looking distance $L_{d}$ acts as the output. Both input and output variables are fuzzified.

**Figure 11:**
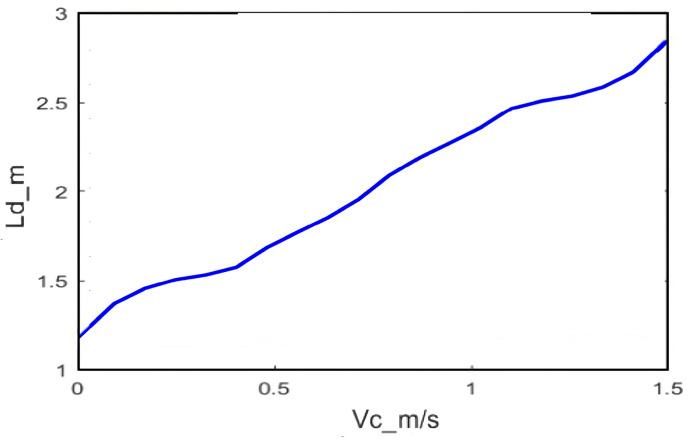
Fuzzy inference curve.

(1) The vehicle speed $v_{C}$ has a basic range of {0 m/s, 1.5 m/s}, and the fuzzy levels for the vehicle speed are defined as very low (VL), low (*L*), moderate (M), high (H), and very high (VH).
(2) The forward-looking distance $L_{d}$ has a basic range of {1 m, 3 m}, and the fuzzy levels for the forward-looking distance are categorized as very near (VN), near (N), moderate (M), far (F), and very far (VF). The input and output variables are fuzzified using the triangular membership function. Drawing from manual driving experience, Table 1 presents the fuzzy control rules, while Figure 11 illustrates the fuzzy inference curve.

#### 2.2.4. Automatic Grain Unloading Control System

**Figure 12:**
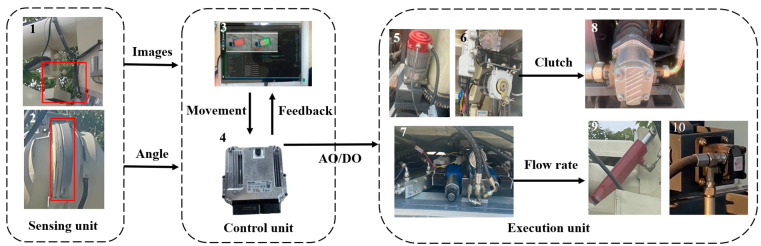
Automatic grain unloading control system. 1. Binocular camera; 2. Angle sensor; 3. Android tablet; 4. Rexroth hydraulic controller; 5. Unloading servo motor; 6. Unloading clutch motor; 7. Unloading hydraulic valve group; 8. Gear pump; 9. Double-acting hydraulic cylinder; 10. Hydraulic motor.

As shown in Figure 12, the automatic grain unloading control system for the tracked grain vehicle comprised three main components: the sensing unit, the control unit, and the execution unit. The sensing unit included a ZED2 binocular camera (Stereolab, Beijing, China), an integrated encoder, and a non-contact angle sensor; the binocular camera was installed in the outlet of the unloading tube to capture downward-facing images. The integrated encoder of the servo motor that controlled the rotation of the unloading tube obtained the unfolding angle of the unloading tube β, while the angle sensor installed under the unloading hydraulic cylinder obtained the lifting angle of the unloading tube γ. Leveraging the geometric relationships in three-dimensional space, the actual landing point of the grain can be projected. The control unit consisted of the Bosch Rexroth BODAS hydraulic controller and an Android tablet, while the execution unit comprised the unloading servo motor, unloading clutch motor, unloading hydraulic valve group, gear pump, double-acting hydraulic cylinder, and hydraulic motor.

**Figure 13:**
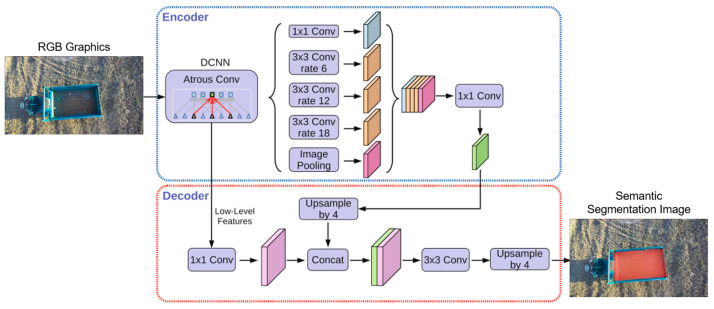
The architecture of the DeepLabv3+ network.

After the combined operation of the combine-harvester and tracked grain vehicle for coordinated grain unloading, they need to return to the field edge and automatically transfer the grain from the grain tank to the wheeled transport vehicle positioned on the field ridge. The automatic recognition and extraction lie at the core of the grain transport vehicle’s unloading process. Considering the variability of grain unloading scenarios and the substantial influence of lighting conditions and dust, this study utilizes semantic segmentation, which achieves pixel-level segmentation, to address potential challenges like blurred boundaries or common misclassification encountered in conventional image classification methods. This methodology facilitates assigning each pixel to a predefined semantic category, enabling adaptability to diverse scales and complex environments. The images captured by the binocular camera were divided into two categories of complete and incomplete unloading regions by semantic segmentation. The DeepLabv3+ network model was employed to extract and identify the unloading region. By combining various rates of dilated convolutions and pyramid pooling, the model captured multi-scale contextual information, enabling faster and more accurate delineation of the contour and shape of the target unloading region. This significantly enhanced the accuracy and real-time performance of the segmentation process. The architecture of the DeepLabv3+ network is illustrated in Figure 13. The extracted unloading region was further divided into multiple sub-regions, with the geometric centers of these sub-regions representing the desired grain landing points.

**Figure 14:**
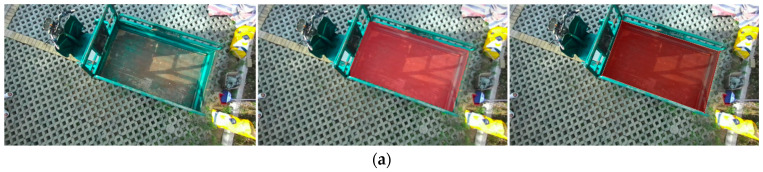

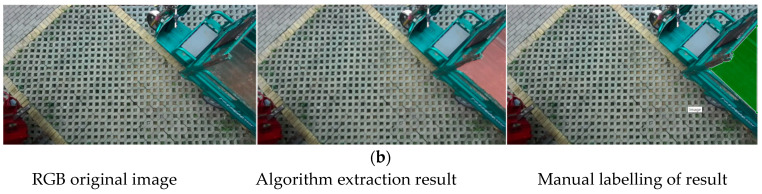
Unloading region identification and extraction: (**a**) Complete unloading region; (**b**) Incomplete unloading region.

Furthermore, to comprehensively account for various factors of variation, multiple adjustments were made to the shooting poses of the grain unloading tube, thereby modifying the shape, size, and distribution of the target unloading area within the camera’s field of view. Detailed annotations were meticulously applied to both complete and incomplete unloading regions, classifying them into two distinct categories. This approach yields a more comprehensive depiction of the shape and size of the target unloading region present in the image data. The collected image data were divided into a training set and a validation set, following a 7:3 ratio, resulting in a dataset comprising a total of 900 images. The image annotation tool, Labelme, was employed for the manual annotation of the unloading region in the images, thereby constructing the training dataset. In this study, the DeepLabv3+ network was chosen as the training model for extracting the unloading region, and its performance was thoroughly evaluated and validated. The extracted results of the unloading region based on the DeepLabv3+ network, post-training, are visually presented in Figure 14.

**Figure 15:**
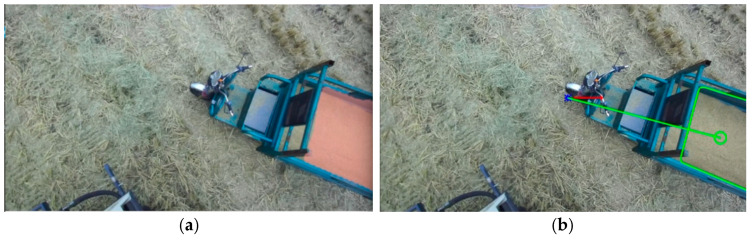
Fitted rectangle of the incomplete unloading area: (**a**) Semantic segmentation effect; (**b**) Fitted rectangle effect.

**Figure 16:**
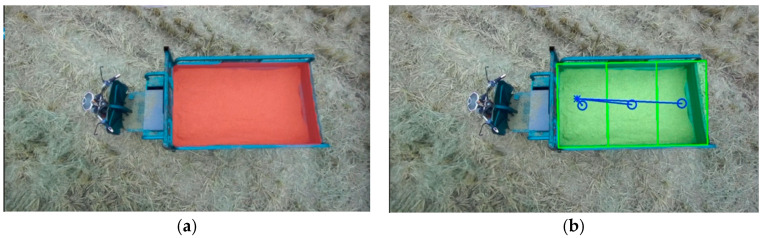
Fitted rectangle of the complete unloading area: (**a**) Semantic segmentation effect; (**b**) Fitted rectangle effect.

**Figure 17:**
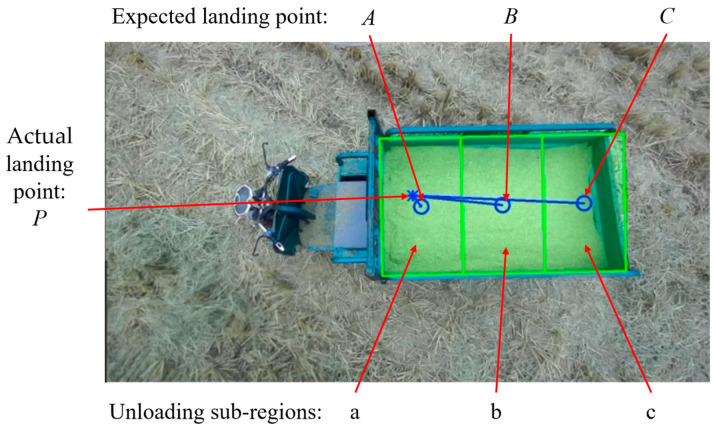
Unloading sub-regions and expected landing points.

Building upon the precise identification and extraction of unloading areas accomplished through the DeepLabv3+ network model, we achieve automated control of the unloading process. As the grain unloading tube initiates its search for the unloading point, the recognized unloading area may be incomplete. To address this, we computed the convex hull of the incomplete region, which serves as a semantic mask. By fitting the convex hull into a polygon, we determined the coordinates of the centroid of this polygon, as depicted in Figure 15, which became the desired unloading point. The rotation motor of the unloading tube was then controlled by adjusting the rotation direction based on the deviation between the calculated actual unloading point and the centroid coordinates of the polygon. This adjustment drove the unloading port towards the desired unloading point until a complete semantic mask of the unloading area was obtained. Once a complete unloading area was recognized, precise positioning of the unloading port was required. The complete unloading area was divided into multiple sub-areas, and the geometric centers of each sub-area were determined as the desired unloading points, along with their corresponding coordinate values. Initially, the semantic mask information of the complete unloading area was transformed into contour information and fitted into the minimum bounding rectangle, as shown in Figure 16, while obtaining the coordinates of its four vertices. Based on the grain bin’s capacity in the transport vehicle, this study uniformly divided the bin into three unloading sub-areas: sub-area a, sub-area b, and sub-area c. By using the coordinates of the four vertices from the bounding rectangle, we calculated the pixel coordinates of the geometric centers of the three sub-areas, representing the desired unloading points. The division of the processed unloading sub-areas and their corresponding desired unloading points are illustrated in Figure 17. Through the partitioning of the unloading sub-areas and the calculation of the unloading point coordinates, precise control of the unloading tube’s movement to each desired unloading point was achieved, resulting in a uniform distribution of the unloading process.

**Figure 18:**
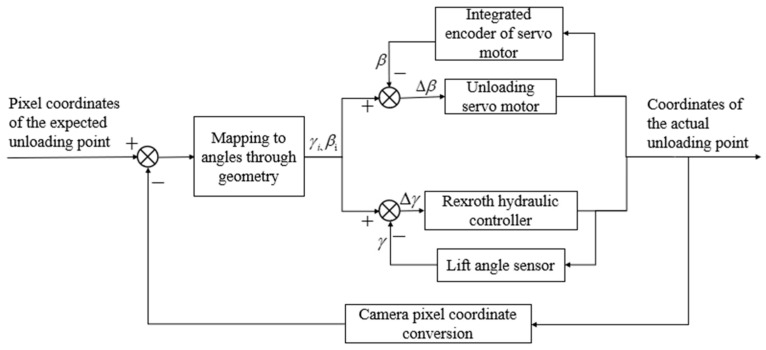
Control model of automatic grain unloading.

The pixel coordinate deviation between the actual and expected positions of the grain landing point in the image was mapped to three-dimensional space to obtain the control angles required for left–right rotation ∆β and up–down swing ∆γ of the unloading chute. These control angles ∆β and ∆γ were used as input signals and passed to the control unit, which outputted CAN signals to the servo motor and hydraulic controller to achieve the left–right rotation and up–down elevation of the unloading chute, so that the actual unloading point of the grain landing was gradually close to the desired position. Once the outlet of the unloading tube was accurately aligned with the target unloading position, the control unit sent a CAN signal to the hydraulic controller, which controlled the rotation of the hydraulic pump and activated the grain unloading stirring auger rotation, initiating the unloading process. The control model of automatic grain unloading is shown in Figure 18.

## 3. Results

**Figure 19:**
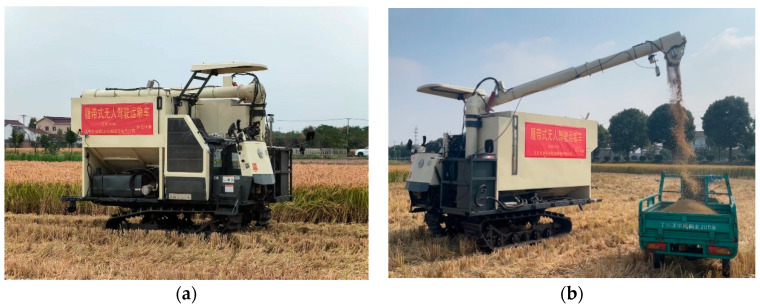
Platform and environment of the trials: (**a**) Experiment environment for automated navigation; (**b**) Experiment site for automatic grain unloading.

In order to verify the stability of the unoccupied driving control system of the tracked grain vehicle, we conducted automatic navigation and automatic unloading experiments in Shiyezhou, Zhenjiang city, Jiangsu Province, and the platform and environment of the experiments are depicted in Figure 19.

### 3.1. Straight-Line Path-Tracking Experiment in the Field

**Figure 20:**
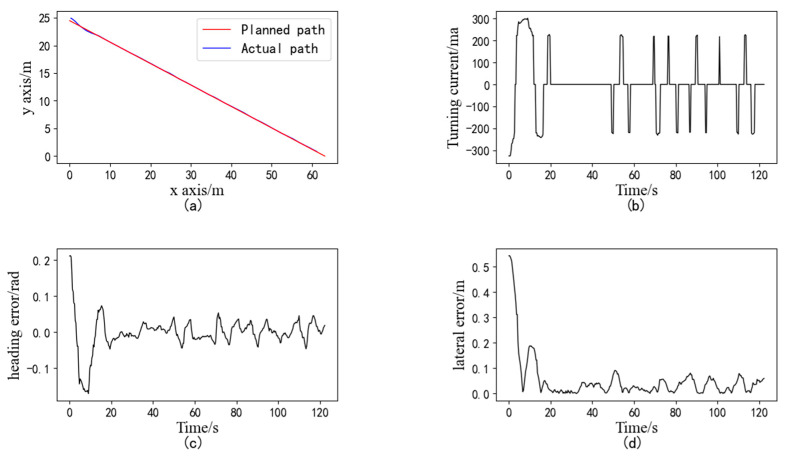
Experiment results of the straight-line path tracking: (**a**) Trajectory of path tracking; (**b**) current of the steering proportional valve; (**c**) heading error; (**d**) lateral error.

The experiment consisted of the following steps: the tracked grain vehicle drove to point B at the head of the field, and the latitude and longitude coordinates of that point were entered into the human–machine interface. The vehicle then reversed and traveled 70 m to point A, where the current coordinates were inputted once again, so that a straight line determined by points A and B was established as the target tracking path. Next, the grain vehicle was started near point A, and the navigation control system was activated for path-tracking control. As the vehicle approached point B, it automatically halted, marking the completion of the straight-line path-tracking experiment. The tracked grain vehicle underwent three repeated experiments at speeds of 0.6 m/s (low speed), 1.0 m/s (medium speed), and 1.4 m/s (high speed), and the initial heading of each experiment was basically parallel to the planned path and in the same direction. The experiment results of the straight-line path tracking of the tracked grain vehicle at a speed of 0.6 m/s are presented in Figure 20. In Figure 20a, the red line represents the planned path, while the blue line represents the actual path of the vehicle. The results indicate an average lateral absolute deviation of 3.14 cm and a maximum deviation of 7.20 cm, satisfying the requirements for unoccupied driving of the grain vehicle [22].

**Figure 21:**
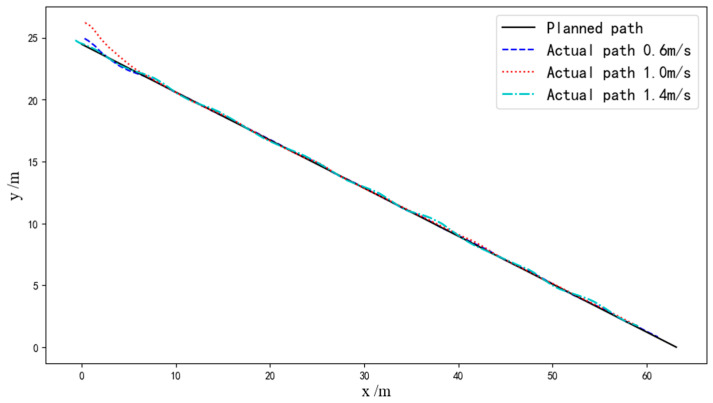
The actual tracking path at different speeds.

**Figure 22:**
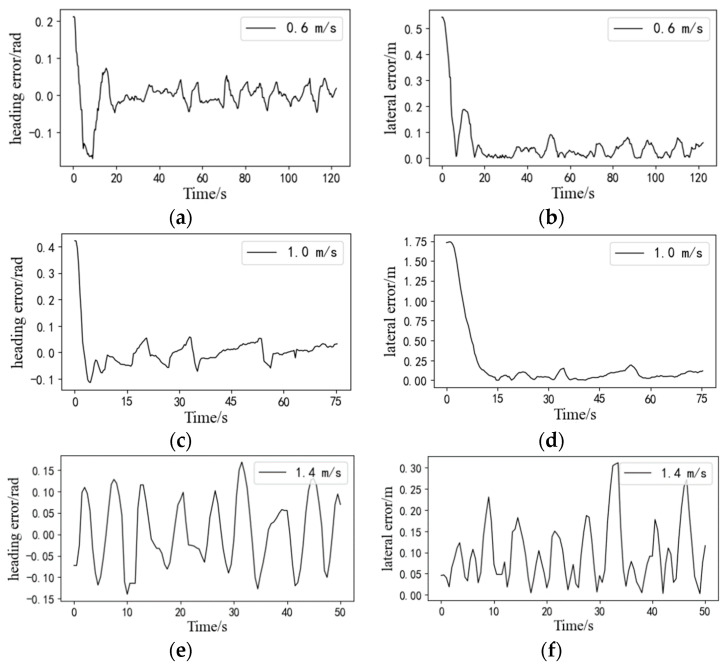
Deviations of straight-line path tracking at different speeds. (**a**) heading error at the speed of 0.6 m/s; (**b**) lateral error at the speed of 0.6 m/s; (**c**) heading error at the speed of 1.0 m/s; (**d**) lateral error at the speed of 1.0 m/s; (**e**) heading error at the speed of 1.4 m/s; (**f**) lateral error at the speed of 1.4 m/s.

The statistical results of all straight-line path-tracking experiments are provided in Table 2. Figure 21 illustrates a comparison of the actual tracking path for a single straight tracking experiment at low, medium, and high speeds. The deviations of the experiments are shown in Figure 22; it is evident that both the average lateral deviation and average heading deviation increased with higher driving speeds. The experiment results demonstrate that the average lateral absolute deviation was 3.14 cm and the standard deviation was 3.47 cm at the low speed of 0.6 m/s; the average lateral absolute deviation was 4.63 cm and the standard deviation was 3.93 cm at the medium speed of 1.0 m/s; and the average lateral absolute deviation was 6.46 cm and the standard deviation was 4.58 cm at the high speed of 1.4 m/s. The accuracy of straight-line path tracking met the requirements for unoccupied driving of the tracked grain vehicle.

### 3.2. Automatic Grain Unloading Experiment

**Figure 23:**
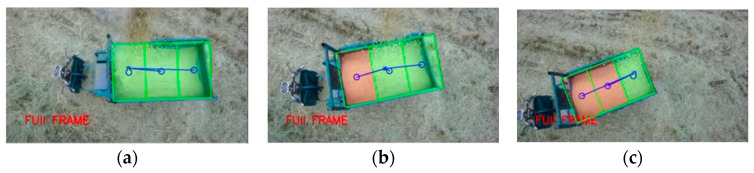
Regulatory effects of the automatic grain unloading experiment. (**a**) Unloading to sub-region a; (**b**) Unloading to sub-region b; (**c**) Unloading to sub-region c.

After the coordinated unloading of the combine harvester and grain vehicle, it was necessary to return to the edge of the field and unload the grain from the grain tank onto the wheeled transport vehicle on the agricultural road. In this experiment, an agricultural tricycle was used to simulate the transport vehicle. The dimensions of the agricultural tricycle’s grain tank were measured as 45 cm × 85 cm × 140 cm, and the grain tank was evenly divided into three unloading sub-regions: a, b, and c. Rice was used as the grain for unloading, and the automatic unloading experiment was carried out for an empty grain tank and a partially piled grain. The relative pixel deviation $\sigma_{i}$ between the actual landing point $P(u_{i},v_{i})$ and the expected landing point $A(u_{a},v_{a})$, $B(u_{b},v_{b})$, $C(u_{c},v_{c})$ of the three unloading sub-regions was calculated to evaluate the accuracy of the unloading positioning regulation system. The deviation results of the automatic grain unloading control experiment are shown in Table 3. The regulatory effects of the automatic grain unloading experiment are illustrated in Figure 23.

The experimental results indicated that the pixel deviation between the actual coordinates and the expected coordinates at each grain unloading point was stable within ±100 pixels, with a maximum deviation of 92 pixels. This demonstrates that the regulation accuracy of the automatic unloading control system is sufficient for practical grain unloading operation.

## 4. Discussion

We presented an unoccupied control system for a tracked grain vehicle, constructed using the Rexroth BODAS controller and an Android tablet. The system comprised a path-tracking control system, employing fuzzy control and pure pursuit algorithms, and an automatic unloading control system, utilizing semantic segmentation technology and the DeepLabv3+ network. Field experiments were conducted to assess the precision and stability of the system. It was observed that the forward-looking distance in the pure pursuit algorithm significantly influenced the tracking accuracy. Therefore, fuzzy control was employed to achieve adaptive adjustments in the forward-looking distance according to the vehicle’s speed. The experiments revealed that the lateral deviation in path tracking increased notably with the vehicle’s speed, but the tracking accuracy of the grain vehicle still met the requirements for unoccupied driving at maximum speed. The automatic unloading control system identified the unloading region through stereo cameras and controlled the movement of the grain unloading tube to the desired unloading point using a controller. The experimental results demonstrated that the system’s control accuracy fulfilled the demands of practical unloading operations.

In comparison to existing automatic driving systems for tracked agricultural machinery, we modified the electro-hydraulic control system of the tracked grain vehicle. Integration of the path-tracking control system and the automatic unloading control system into an Android tablet, with a single Rexroth BODAS controller, facilitated coordination between the two systems and simplified the control equipment. The fuzzy pure pursuit control method employed in the path-tracking control system not only achieved adaptive adjustments in the forward-looking distance but also enhanced the tracking accuracy of the tracked grain vehicle at different speeds. The automatic unloading control system, employing semantic segmentation algorithms, improved identification accuracy compared to simple envelope line extraction and achieved higher recognition efficiency than three-dimensional point cloud-filling techniques, enabling faster control of the grain unloading tube to the desired unloading point.

The path-tracking control system of the tracked grain vehicle primarily regulated lateral deviation by controlling the steering current value. However, when operating in conjunction with a harvester, it is necessary to synchronize the forward speed of the grain vehicle with that of the harvester. Therefore, incorporating a longitudinal tracking control algorithm is crucial for accurately controlling the grain vehicle’s forward speed. Additionally, the present study solely considered the impact of speed on control accuracy, while other factors such as field terrain conditions need to be comprehensively evaluated to further optimize the path-tracking control algorithm and enhance tracking accuracy. The automatic unloading control system is susceptible to interference from background factors during the identification of unloading regions, resulting in misidentification and edge fluctuation issues. Future enhancements could involve refining the DeepLabv3+ semantic segmentation algorithm or exploring alternative segmentation algorithms to improve recognition accuracy for target unloading regions. Furthermore, during the control of the grain unloading tube, overshoot phenomena occasionally occur, necessitating optimization of the closed-loop control method to heighten control accuracy and efficiency.

## 5. Conclusions

This paper designed and developed an unoccupied control system for a tracked grain vehicle and conducted experiments on automatic navigation control and automatic unloading control in the field. The main conclusions are as follows:

(1) Based on the exclusive version of the combine harvester chassis of the WORLD Company, we designed and manufactured an unoccupied tracked grain vehicle with a loading capacity of 4.5 m^3^ with its accompanying unloading hydraulic system. The design of the electronic control system that combined manual and automatic operation for the chassis’ walking and unloading mechanisms was realized on the basis of the BODAS hydraulic controller.
(2) We employed a hybrid A* algorithm to plan the travelling path of the tracked grain vehicle and devised a path-tracking control system by combining the fuzzy control and pure pursuit algorithms. Field experiments were conducted to evaluate the system’s performance at speeds of 0.6 m/s, 1.0 m/s, and 1.4 m/s. The results revealed that at a speed of 0.6 m/s, the average lateral deviation during straight path tracking was 3.14 cm, with a standard deviation of 3.47 cm. At a speed of 1.0 m/s, the average lateral deviation increased to 4.63 cm, with a standard deviation of 3.93 cm. When traveling at 1.4 m/s, the average lateral deviation further increased to 6.46 cm, with a standard deviation of 4.58 cm. These results demonstrate that the grain transport vehicle meets the requirements for unoccupied driving.
(3) Utilizing binocular vision cameras and semantic segmentation technology, we designed an automatic unloading control system with grain tank recognition and unloading regulation capabilities. Field experiments were conducted in harvested rice fields to evaluate the system’s performance. The results indicated that the maximum absolute pixel deviation between the actual unloading point and the desired unloading point was 92 pixel values. This demonstrates that the control accuracy of the automatic unloading control system can meet the use of actual grain unloading operations.

## Figures and Tables

**Table 1:** Fuzzy control rules.

$\mathrm{If}\;v_{C}$	VL	*L*	M	H	VH
Then $L_{d}$	VN	N	M	F	VF

**Table 2:** Experiment results of tracking accuracy at different speeds.

Driving Speed	Experiment Number	Average Lateral Deviation (cm)	Average Heading Deviation (rad)
0.6 m/s	1	3.14	0.022
	2	3.22	0.024
	3	3.06	0.018
	on average	3.14	0.021
1.0 m/s	1	4.59	0.032
	2	4.63	0.038
	3	4.47	0.028
	on average	4.56	0.033
1.4 m/s	1	6.52	0.063
	2	6.46	0.052
	3	6.66	0.056
	on average	6.55	0.057

**Table 3:** Pixel coordinate deviation results of each unloading point.

Unloading Point	Coordinates of the Expected u-Axis	Coordinates of the Actual u-Axis	Coordinates of the Expected v-Axis	Coordinates of the Actual v-Axis	$\mathrm{Pixel}\;\mathrm{Deviation}\;\sigma_{i}$
A	746	801	203	238	66
B	728	784	254	326	92
C	597	652	281	347	86

## Data Availability

The dataset can be accessed at https://pan.baidu.com/s/1u0ME3RyvrZzCxvF1kwK61g?pwd=0425.
